# Pheonolic Compounds from the Fruits of *Viburnum sargentii* Koehne

**DOI:** 10.3390/molecules200814377

**Published:** 2015-08-06

**Authors:** Yang Xie, Jing Wang, Yan-Mei Geng, Zhi Zhang, Yan-Fei Qu, Guang-Shu Wang

**Affiliations:** School of Pharmaceutical Sciences, Jilin University, Changchun 130021, China; E-Mails: 13514486691@163.com (Y.X.); 13578752256@163.com (J.W.); gengym0102@163.com (Y.-M.G.); zz4548209@163.com (Z.Z.); wdcq2511@163.com (Y.-F.Q.)

**Keywords:** *Viburnum sargentii*, phenolic glycoside, monoterpene, epicatechin, quercetin

## Abstract

Seven phenolic compounds were isolated from the fruits of *Viburnum sargentii* Koehne by silica gel column chromatography and preparative HPLC. On the grounds of chemical and spectroscopic methods, their structures were identified as (−)-Epicatechin (**1**), 5,7,4′-trihydroxy-flavonoid-8-*C*-β-d-glucopyranoside (**2**), 1-(4-hydroxy-3-methoxyphenyl)-2-[4-(3-α-l-rhamnopyranoxypropyl)-2-methoxyphenoxy]-1,3-propane-diol (erythro) (**3**), 1-(4-hydroxy-3-methoxyphenyl)-2-[4-(3-α-l-rhamnopyranoxypropyl)-2-methoxyphenoxy]-1,3-propanediol (threo) (**4**), (*R*)-4-hydroxylphenol *O*-(6-*O*-oleuropeoyl)-β-d-glucopyranoside (**5**), (*R*)-3-methoxy-4-hydroxylphenol *O*-(6-*O*-oleuropeoyl)-β-d-glucopyranoside (**6**), quercetin-3-*O*-rutinoside (**7**). Compounds **5** and **6** are new monoterpene phenolic glycosides, compounds **1**, **3** and **4** were isolated from the *Viburnum* genus for the first time, and compounds **2** and **7** from the *Viburnum* *sargentii* Koehne for the first time. Compounds **1**–**7** were also assayed for their antioxidant activities with DPPH free radicals.

## 1. Introduction

**Figure 1:**
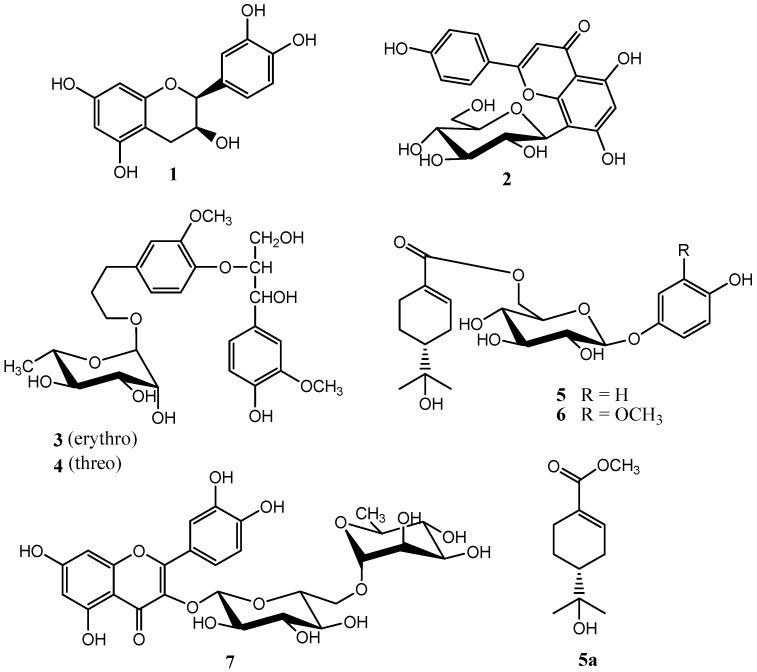
Chemical structures of compounds **1**–**7**.

*Viburnum* *sargentii* koehne, a deciduous shrub of *Caprifoliaceae*, is widely distributed in the northeast and northwest regions of China. As traditional Chinese medicines, the branches are used for rheumatoid arthritis and traumatic injuries, the leaves for boils, ringworm and skin itching, and the fruits for cough [1]. Although there are a number of the pharmacological studies of the fruits of *Viburnum sargentii* [2,3], the isolation and structure identification have not been investigated in detail. As one part of our *Caprifoliaceae* studies, the isolation and structure identification of chemical constituents from the fruits of *Viburnum sargentii* Koehne have been carried out, and we report the isolation and identification of two new monoterpene phenolic glycosides (**5** and **6**), together with five phenolic compounds **1**, **2**, **3**, **4** and **7** in the present study (Figure 1).

## 2. Results and Discussion

**Figure 2:**
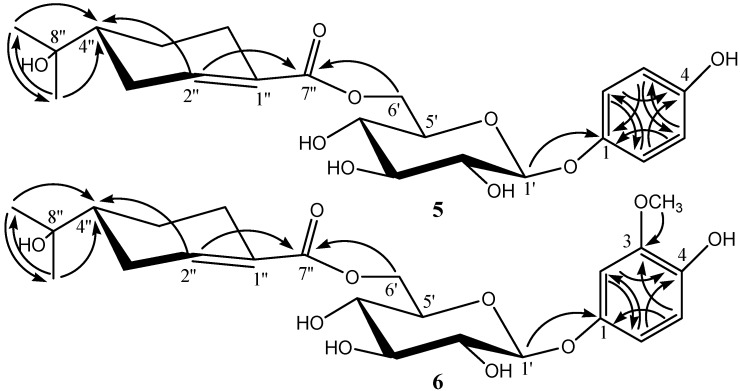
The Key HMBC Correlation of compound **5** and **6** (arrows point from proton to carbon).

Compound **5** was obtained as colorless needles. Its HRESIMS displayed a [M + H]^+^ ion peak at *m*/*z* 439.1957 (calcd for C_22_H_31_O_9_, 439.1968), indicating the molecular formula C_22_H_30_O_9_. Acidic hydrolysis of **5** yielded d-glucose as a sugar residue. The ^1^H- and ^13^C-NMR spectra showed two 2H doublets (δ_H_ 6.83 (2H, d, *J* = 8.7 Hz), 7.03 (2H, d, *J* = 8.7 Hz)) and signals of six symmetric aromatic C-atoms at δ_C_ 154.3 (C), 152.1(C), 119.5 (2 CH), and 117.4 (2 CH), arising from a symmetrically 1,4-*O*-disubstituted benzene ring moiety, a set of signals characteristic of β-d-glucopyranosyl moiety (anomeric H-atom signal at δ_H_ 4.88 (d, *J* = 7.5 Hz)). The ^13^C-NMR and DEPT spectra showed ten carbon signals comprising one carboxylic (δ_C_ 168.2), one trisubstituted double bond (δ_C_ 142.2, 131.5), two methyl (δ_C_ 27.1, 26.6), three methylene (δ_C_ 29.1, 26.9, 24.9), one methine (δ_C_ 45.7), and one oxygen-bearing quaternary carbon (δ_C_ 72.2). These observations suggested the presence of an oleuropeic acid unit [4,5]. The positions of the oleuropeoyl ester and glycosidic linkages in **5** were established by 2D-NMR experiments. In the HMBC spectrum of **5**, the glucosyl CH_2_ (6′) (δ_H_ 4.60, 4.22) correlated with the oleuropeoyl carboxylic C-atom C (7″) (δ_C_ 168.2), and the glucosyl H-C (1′) (δ_H_ 4.88) with and C (1) (δ_C_ 152.1) of 1,4-*O*-disubstituted benzene ring moiety. The full assignments of all protons and carbons were preformed through the correlations in 2D-NMR spectra (^1^H-^1^H COSY, HMQC and HMBC) of **5**. All the data of ^1^H-, ^13^C-, and HMBC-NMR of compound **5** see Table 1, and key correlations and the structure of compound **5** see Figure 2. Methanolysis of **5** with MeONa in MeOH afforded **5a** which was identified as (*R*)-oleuropeic acid methyl ester by comparing ${\left[\alpha \right]}_{D}^{25}$ and ^1^H- and ^13^C-NMR spectra data with the reported [4,5]. Based on the above evidence, the structure of **5** was determined to be (*R*)-4-hydroxylphenol *O*-(6-*O*-oleuropeoyl)-β-d-glucopyranoside.

**Table 1:** ^1^H-NMR (methanol-*d*_4_, 400 MHz), ^13^C-NMR (methanol-*d*_4_, 100 MHz) and HMBC data of compound **5** and **6** (TMS as the internal standard, δ in ppm *J* in Hz).

No.	5			6		
	δ_C_	δ_H_ *J* (Hz)	HMBC (H→C)	δ_C_	δ_H_ *J* (Hz)	HMBC (H→C)
1	152.1			153.0		
2	119.5	7.03 d (8.7)	154.3, 119.5	104.5	6.80 br. s	143.4, 110.4
3	117.4	6.83 d (8.7)	152.1, 117.4	149.2		
3-OCH_3_				56.8	3.90 s	149.2
4	154.3			143.4		
5	117.4	6.83 d (8.7)	152.1, 117.4	116.2	6.75 d (8.2)	153.0, 149.2
6	119.5	7.03 d (8.7)	119.5, 154.3	110.4	6.65 d (8.2)	104.5, 143.4
1′	103.3	4.88 d (7.5)	168.2	103.6	4.83 d (7.8)	153.0
2′	75.2	3.29 m		75.2	3.51 m	
3′	78.4	3.46 m		78.2	3.53 m	
4′	72.2	3.36 m		72.3	3.43 m	
5′	75.6	3.75 m		75.8	3.71 m	
6′α	65.6	4.60 d (11.5)	168.2	65.2	4.59 d (11.5)	168.2
6′β		4.22 dd (11.5, 7.7)	168.2		4.32 dd (11.5, 7.3)	168.2
1″	131.5			131.5		
2″	142.2	7.04 br. s	168.2, 29.1, 45.7	141.8	7.09 br. s	168.2, 45.4
3″α	29.1	2.48 d (16.5)		28.9	2.42 d (17.5)	
3″β		2.20 m			2.12 m	
4″	45.7	1.63 m		45.4	1.65 m	
5″α	24.9	2.16 m		24.8	2.12 m	
5″β		1.32 m			1.32 m	
6″α	26.9	2.59 d (16.0)		26.7	2.51 d (16.4)	
6″β		2.26 m			2.21 m	
7″	168.2			169.0		
8″	72.2			73.2		
9″	27.1	1.28 s	45.7, 72.2, 26.6	27.3	1.38 s	45.4, 73.2, 26.8
10″	26.6	1.28 s	45.7, 72.2, 27.1	26.8	1.28 s	45.4, 73.2, 27.3

Note: The assignments were based on DEPT, HMQC, ^1^H-^1^H COSY, and HMBC experiments.

Compound **6** was obtained as colorless needles. Its molecular formula C_23_H_3__2_O_10_ was elucidated from the HRESIMS *m*/*z* 469.2086 [M + H]^+^ (calcd for C_23_H_33_O_10_, 469.2074). Acidic hydrolysis of **6** also yielded d-glucose as a sugar residue. The ^1^H- and ^13^C-NMR spectra of **6** were similar to those of **5**, except for the signals of benzene ring moiety. The ^1^H- and ^13^C-NMR spectra showed a typical three proton ABX aromatic spin system at δ_H_ 6.75 (1H, d, *J* = 8.2 Hz), 6.65 (1H, d, *J* = 8.2 Hz), 6.80 (1H, br. s) and six aromatic C-atom signals at δ_C_ 153.0 (C), 149.2 (C), 143.4 (C), 116.2 (CH), 110.4 (CH) and 104.5 (CH), as well as one signal from a MeO group [δ_H_ 3.90 (s, 3H), δ_C_ 56.8) ], suggesting that there is a 1,3,4-*O*-trisubstituted benzene ring moiety in compound **6** in stead of a 1,4-*O*-disubstituted benzene ring moiety, and one additional methoxy group. The positions of the oleuropeoyl ester, glycosidic linkages and MeO group in **6** were also established by 2D-NMR experiments. In the HMBC spectrum of **6**, the glucosyl CH_2_ (6′) (δ_H_ 4.59, 4.32) correlated with the oleuropeoyl carboxylic C-atom C (7″) (δ_C_ 169.0), the glucosyl H-C (1′) (δ_H_ 4.83) with C (1) (δ_C_ 153.0) of the 1,3,4-*O*-trisubstituted benzene ring moiety, and the MeO group δ_H_ 3.90 correlated with C (3) (δ_C_ 149.2) of 1,3,4-*O*-trisubstituted benzene ring moiety. The full assignments of all protons and carbons were preformed through the correlations in 2D-NMR spectra (^1^H-^1^H COSY, HMQC and HMBC) of **6**. All the data of ^1^H-, ^13^C-, and HMBC-NMR of compound **6** see Table 1, and key correlations and the structure of compound **6** see Figure 2. Methanolysis of **6** also afforded **5a**. Thus, the structure of **6** was determined to be (*R*)-3-methoxy-4-hydroxylphenol *O*-(6-*O*-oleuropeoyl)-β-d-glucopyranoside.

Using similar methods as described above, compounds **1**–**4** and **7** were identified as (−)-Epicatechin (**1**) [6], 5,7,4′-trihydroxy-flavonoid-8-*C*-β-d-glucopyranoside (**2**) [7], 1-(4-hydroxy-3-methoxy-phenyl)-2-[4-(3-α-l-rhamnopyranoxypropyl)-2-methoxyphenoxy]-1,3-propane-diol (erythro) (**3**) [8], 1-(4-hydroxy-3-methoxyphenyl)-2-[4-(3-α-l-rhamnopyranoxypropyl)-2-methoxyphenoxy]-1,3-propanediol (threo) (**4**) [8], quercetin-3-*O*-rutinoside (**7**) [9].

Compounds **1**–**7** were next assayed for their antioxidant activity with DPPH free radicals, and the results are shown in Table 2. The data proved that (−)-Epicatechin showed strongest antioxidant activity.

**Table 2:** IC_50_ values of compounds **1**–**7**.

Compound	IC_50_ (µg·mL^−1^)
**1**	9.85 ± 0.5
**2**	357.1 ± 6.2
**3**	520.9 ± 7.6
**4**	522.3 ± 8.1
**5**	610.8 ± 6.1
**6**	620.1 ± 7.3
**7**	37.5 ± 0.3

Note: All values are averages of at least three runs in Table 2.

## 3. Experimental Section

### 3.1. General Information

NMR spectra were recorded on a Bruker AV-400 spectrometer (Bruke Corporation, Faellanden, Switzerland). UV Spectra were recorded on a Shimadzu UV-2401A spectrometer (Shimadzu Corporation, Kyoto, Japan). HR-ESI-MS were recorded on a Bruker microOTOF-Q II mass spectrometer (Bruke Corporation, Bremen, Germany). Optical rotations were measured with a HORIBA SEPA-300 high-sensitive polarimeter (Horiba Ltd, Kyoto, Japan). Melting points (m.p.) were measured with a X-4 Microscopic melting point apparatus (Shanghai Hui Tong Optical Instrument Co., Ltd, Shanghai, China). HPLC was performed Shimadzu LC-10A with a SPD-10A detector (Shimadzu Corporation, Kyoto, Japan) and Gemini 5μ C18 110A column (250 mm × 10.00 mm, 5 μm, flow rate: 3.0 mL/min, Phenomenex, Torrance, CA, USA). GC was performed an Agilent 7820A gas chromatograph with a quartz capillary column (30 mm × 0.32 mm × 0.25 μm, Agilent Technolgies Inc., Santa Clara, CA, USA); detection, FID. Column chromatography was performed on silica gel (200–300 mesh, Qingdao Marine Chemical Inc., Qingdao, China), D101 polyporous resin (Tianjin Pesticide Co., LTD., Resin Branch, Tianjin, China), polyamide (80–120 mesh, Taizhou Luqiao Sijia Biochemical Plastic Factory, Taizhou, China) and MCI-gel CHP-20P (75–150 μm; Mitsubishi Chemical Co., Tokyo, Japan). TLC was performed on glass precoated silica gel GF_254_ plates (Qingdao Haiyang Chemical Co., Ltd, Qingdao, China), detection under UV light or by spraying with 10% H_2_SO_4_ in 95% EtOH followed by heating. The bioactivities were measured on a DNM-9602 Enzyme immunoassay spectrophotometer (Beijing, China), using 1,1-diphenyl-2-picrylhydrazyl free radical (DPPH) (Sigma-Aldrich, Shanghai, China). The fruits of *Viburnum sargentii* Koehne were collected in Changchun District of Jilin Province, China. They were identified by Jing-min Zhang of the School of Pharmaceutical Sciences, Jilin University. 

### 3.2. Extraction and Isolation

The fresh fruits of *V. sargentii* (8 kg) were extracted with 70% aqueous ethanol at room temperature (3 L × 10 L, weekly). The extracts were concentrated under reduced pressure and then subjected to D101 polyporous resin column chromatography eluated with H_2_O, 10% aqueous ethanol, 30% aqueous ethanol, 60% aqueous ethanol and 95% aqueous ethanol. The eluate of 30% ethanol was chromatographed over silica gel, eluting with CHCl_3_–EtOAc–MeOH–H_2_O (3.5:1.2:4:1.2, *v*/*v*, lower layer), to afford four fractions, Frs. 1–4. Compound **1** (521 mg) was recrystallized from Fr. 1, Fr. 2 and 4 were further chromatographicly separated by gradient elution with MeOH–H_2_O (from 0% to 65%, 5% a time, *v*/*v*), and recrystallization of compound **2** (45 mg) were obtained from Fr. 2 and compound **7** (100 mg) from Fr. 4. Fr. 3 was further subjected to silica gel column chromatography eluting with CHCl_3_–EtOAc–MeOH–H_2_O (3.0:1.2:4:1.2, *v*/*v*, lower layer) to afford three subfractions, Frs. 3a–c. They were further isolated by semi-preparative RP-HPLC using acetonitrile–H_2_O as the mobile phase. Compound **3** (47 mg) and **4** (101 mg) were obtained from Fr. 3a with gradient elution (10%–15% acetonitrile from 0.00–10.00 min, 15%–18% acetonitrile from 10.00–20.0 0 min, 18% acetonitrile from 20.00–50.00 min), and by using 18% acetonitrile as the mobile phase compound **5** (62 mg) from Fr. 3b and compound **6** (57 mg) from Fr. 3c.

Compound **1**: Pale amorphous powder, yielded a positive reaction to FeCl_3_ reagent. mp 234–236 °C. ${\left[\alpha \right]}_{D}^{25}$ −56.8 (*c* 1.0, MeOH). UV (MeOH), λ_max_ 217, 280 nm. HRESIMS *m*/*z* 291.0877 [M + H]^+^ (calcd for C_15_H_15_O_6_, 291.0869). ^1^H-NMR (DMSO-*d*_6_, 400 MHz) δ: 2.48 (1H, dd, *J* = 16.0, 3.6 Hz, H-4a), 2.68 (1H, dd, *J* = 16.0, 4.8 Hz, H-4b), 4.01(1H, m, H-3), 4.65 (1H, d, *J* = 4.8 Hz, H-2), 5.89 (1H, d, *J* = 2.0 Hz, H-8), 5.72 (1H, d, *J* = 2.0 Hz, H-6), 6.64 (1H, dd, *J* = 8.0, 1.6 Hz, H-6′), 6.67 (1H, d, *J* = 8.0 Hz, H-5′), 6.89 (1H, d, *J* = 1.6 Hz, H-2′). ^13^C-NMR (DMSO-*d*_6_,100 MHz) δ: 78.0 (C-2), 64.9 (C-3), 28.2 (C-4), 156.5 (C-5), 94.1 (C-6), 156.2 (C-7), 95.1 (C-8), 155.8 (C-9), 98.5 (C-10), 130.6 (C-1′), 114.9 (C-2′), 144.5 (C-3′), 144.4 (C-4′), 114.7 (C-5′), 117.9 (C-6′).

Compound **2**: Yellow amorphous powder, yielded a positive reaction to FeCl_3_ reagent. mp 238–240 °C. UV (MeOH), λ_max_ 268, 339. HRESIMS *m*/*z* 433.1126 [M + H]^+^ (calcd for C_21_H_21_O_10_, 433.1135). ^1^H-NMR (DMSO-*d*_6_, 400 MHz) δ: 8.02 (2H, d, *J* = 8.0 Hz, H-2′,6′), 6.89 (2H, d, *J* = 8.0 Hz, H-3′,5′), 6.77 (1H, s, H-3), 6.28 (1H, s, H-6), 4.69 (1H, d, *J* = 8.0Hz, Glu-H-1). ^13^C-NMR (DMSO-*d*_6_, 100 MHz) δ: (C-7), 104.6 (C-8), 156.0 (C-9), 104.0 (C-10), 121.6 (C-1′), 128.9 (C-2′), 115.8 (C-3′), 160.3 (C-4′), 115.8 (C-5′), 128.9 (C-6′), 73.3 (Glu-1), 70.8 (Glu-2), 78.6 (Glu-3), 70.5 (Glu-4), 81.8 (Glu-5), 61.3 (Glu-6).

Compound **3**: Pale amorphous powder, yielded a positive reaction to FeCl_3_ reagent. mp 190–192 °C. ${\left[\alpha \right]}_{D}^{25}$ −26.9 (*c* 0.30, MeOH). UV (MeOH), λ_max_ 228, 280. HRESIMS *m*/*z* 525.2328 [M + H]^+^ (calcd for C_26_H_37_O_11_, 525.2336). ^1^H-NMR (methanol-*d*_4_, 400 MHz) δ: 7.10 (1H, s, H-2′), 6.92 (2H, d, *J* = 8.0 Hz, H-6′,5), 6.88 (1H, s, H-2), 6.83 (1H, d, *J* = 8.0 Hz, H-5′), 6.75 (1H, d, *J* = 8.0 Hz, H-6), 4.92 (1H, d, *J* = 7.6 Hz, H-7′), 4.73 (1H, s, Rha-H-1), 4.38 (1H, m, H-8′), 3.94 (1H, m, H-9′a), 3.83 (1H, m, H-9′b), 3.74 (1H, m, H-9a), 3.46 (1H, m, H-9b), 2.72 (2H, m, H-7), 1.96 (2H, m, H-8), 3.90 (3H, s, 3′-OCH_3_), 3.88 (3H, s, 3-OCH_3_), 1.32 (3H, d, *J* = 6.4 Hz, Rha-H-6). ^13^C-NMR (methanol-*d*_4_, 100 MHz) δ: 138.1 (C-1), 114.3 (C-2), 152.2 (C-3), 147.5 (C-4), 119.9 (C-5), 122.2 (C-6), 33.3 (C-7), 32.7 (C-8), 68.0 (C-9), 134.4 (C-1′), 112.1 (C-2′), 149.0 (C-3′), 147.3 (C-4′), 116.0 (C-5′), 121.2 (C-6′), 74.4 (C-7′), 86.9 (C-8′), 62.4 (C-9′), 56.8 (3′-OCH_3_), 56.7 (3′-OCH_3_), 102.0 (Rha-1), 72.8 (Rha-2), 72.6 (Rha-3), 74.3 (Rha-4), 70.1 (Rha-5), 18.3 (Rha-6).

Compound **4**: Pale amorphous powder, yielded a positive reaction to FeCl_3_ reagent. mp 186–188 °C. ${\left[\alpha \right]}_{D}^{25}$ −29.5 (*c* 0.30, MeOH). UV (MeOH), λ_max_ 230, 280. HRESIMS *m*/*z* 525.2325 [M + H]^+^ (calcd for C_26_H_37_O_11_, 525.2336). ^1^H-NMR (methanol-*d*_4_, 400 MHz) δ: 7.11 (1H, s, H-2′), 7.07 (H, d, *J* = 8.0 Hz, H-5), 6.95 (1H, d, *J* = 8.0Hz, H-6′), 6.94 (1H, s, H-2), 6.85 (1H, d, *J* = 8.0 Hz, H-5′), 6.80 (1H, d, *J* = 8.0 Hz, H-6), 4.96 (1H, d, *J* = 6.8 Hz, H-7′), 4.73(1H, s, Rha-H-1), 4.30 (1H, m, H-8′), 3.80 (1H, m, H-9′a), 3.74 (1H, m, H-9′b), 3.54 (1H, m, H-9a), 3.45 (1H, m, H-9b), 2.74 (2H, m, H-7), 1.97 (2H, m, H-8), 3.95 (3H, s, 3′-OCH_3_), 3.91 (3H, s, 3-OCH_3_), 1.32 (3H, d, *J* = 6.0 Hz, Rha-H-6). ^13^C-NMR (methanol-*d*_4_, 100 MHz) δ: 138.2 (C-1), 114.2 (C-2), 152.0 (C-3), 148.0 (C-4), 119.9 (C-5), 122.4 (C-6), 33.3 (C-7), 32.7 (C-8), 68.0 (C-9), 134.1 (C-1′), 112.0 (C-2′), 149.1 (C-3′), 147.5 (C-4′), 116.2 (C-5′), 121.1 (C-6′), 74.5 (C-7′), 88.0 (C-8′), 62.2 (C-9′), 56.9 (3′-OCH_3_), 56.7 (3′-OCH_3_), 102.0 (Rha-1), 72.8 (Rha-2), 72.7 (Rha-3), 74.3 (Rha-4), 70.1 (Rha-5), 18.3 (Rha-6).

Compound **5**: colorless needles (MeOH), yielded a positive reaction to FeCl_3_ reagent. mp 198–200 °C, ${\left[\alpha \right]}_{D}^{25}$ −17.5 (*c* 1.0, MeOH). UV (MeOH), λ_max_ 210, 261nm. HRESIMS *m*/*z* 439.1957 [M + H]^+^ (calcd for C_22_H_31_O_9_, 439.1968). ^1^H-NMR (methanol-*d*_4_, 400 MHz), see Table 1; ^13^C-NMR (methanol-*d*_4_, 100 MHz), see Table 1.

Compound **6**: colorless needles (MeOH), yielded a positive reaction to FeCl_3_ reagent. mp 190–192 °C, ${\left[\alpha \right]}_{D}^{25}$ −24.5 (*c* 0.8, MeOH). UV (MeOH), λ_max_ 215, 269 nm. HRESIMS *m*/*z* 469.2086 [M + H]^+^ (calcd for C_23_H_33_O_10_, 469.2074). ^1^H-NMR (methanol-*d*_4_, 400 MHz), see Table 1; ^13^C-NMR (methanol-*d*_4_, 100 MHz), see Table 1.

Compound **7**: Yellow amorphous powder, yielded a positive reaction to FeCl_3_ reagent. mp 185–187 °C. UV (MeOH), λ_max_ 259, 359. HRESIMS *m*/*z* 611.1620 [M + H]^+^ (calcd for C_27_H_31_O_16_, 611.1612). ^1^H-NMR (DMSO-*d*_6_, 400MHz) δ: 6.19 (1H, s, H-6), 6.38 (1H, s, H-8), 7.53 (1H, d, *J* = 1.6 Hz, H-2′), 6.84 (1H, d, *J* = 8.0 Hz, H-5′), 7.54 (1H, dd, *J* = 8.0, 1.6 Hz, H-6′), 5.34 (1H, d, *J* = 6.8 Hz, Glc-H-1), 4.39 (1H, s, Rha-H-1), 1.00 (3H, d, *J* = 6.0 Hz, Rha-H-6). ^13^C-NMR (DMSO-*d*_6_, 100 MHz) δ: 156.4 (C-2), 133.3 (C-3), 177.3 (C-4), 161.2 (C-5), 98.6 (C-6), 164.1 (C-7), 93.5 (C-8), 156.5 (C-9), 103.9 (C-10), 121.1 (C-1′), 115.2 (C-2′), 144.7 (C-3′), 148.4 (C-4′), 116.2 (C-5′), 121.5 (C-6′), 101.2 (Glc-1), 74.0 (Glc-2), 76.4 (Glc-3), 70.0 (Glc-4), 75.9 (Glc-5), 66.9 (Glc-6), 100.7 (Rha-1), 70.5 (Rha-2), 70.3 (Rha-3), 71.8 (Rha-4), 68.2 (Rha-5), 17.7 (Rha-6).

### 3.3. Acid Hydrolysis of ***5*** and ***6***

Compounds **5** and **6** (each 6 mg) were hydrolyzed with 1.5 N HCl (2 mL) at 80 °C for 5 h. The mixture was then neutralized with NaOH (1 N). The mixture was passed through MCI-gel CHP-20P, developing with H_2_O. The H_2_O eluate was evaporated to dryness. The dry powders were dissolved in pyridine (2 mL), l-cysteine methyl ester hydrochloride (1.5 mg) was added, and the mixture was heated at 60 °C for 1 h. Trimethylsilylimidazole (1.5 mL) was added, and the mixture was heated at 60 °C for another 0.5 h. An aliquot (4 μL) of the supernatant was subjected to GC analysis under the following conditions: column temp 180–280 °C at 3 deg/min, carrier gas N_2_ (1 mL/min), injector and detector temp 250 °C, split ratio 1:50. The configurations of d-gluose for compounds **5** and **6** were determined by comparison of the retentions times of the corresponding derivatives with standard d-glucose (retention time: 19.208 min), respectively.

### 3.4. Methanolysis of ***5*** and ***6***

A solution of **5** (12 mg) in 0.02 M NaOMe–MeOH (2 mL) was kept standing at room temperature for 12 h. The solution was then subjected to MCI-gel CHP-20P column chromatography, eluting with H_2_O, 60% and 100% MeOH to give (*R*)-methyl oleuropeic acid methyl ester (**5a**) (3.0 mg): colorless oil; ${\left[\alpha \right]}_{D}^{25}$ +65.5 (*c* 0.2, CHCl_3_); ^1^H-NMR (CDCl_3_, 400MHz) δ 7.06 (1H, m, H-2), 2.30, 2.00 (m, H-3), 1.53 (m, H-4), 2.01, 1.22 (m, H-5), 2.51, 2.12 (m, H-6), 1.20 (3H, s, H-9), 1.21 (3H, s, H-10), 3.72 (3H, s, OCH_3_); ^13^C-NMR (CDCl_3_, 100MHz) δ 130.2 (C-1), 139.9 (C-2), 27.4 (C-3), 44.5 (C-4), 23.4 (C-5), 25.1 (C-6), 167.8 (C-7), 72.2 (C-8), 27.4 (C-9), 26.6 (C-10), 51.6 (OCH_3_). Similar methanolysis of **6** also gave **5a** (${\left[\alpha \right]}_{D}^{25}$ +62.7 (*c* 0.18, CHCl_3_)).

### 3.5. Bioactivity Assay

The antioxidant activities of compounds **1**–**7** were assessed according to their DPPH (1,1-diphenyl-2-picrylhydrazyl free radical, Sigma-Aldrich, Shanghai, China) scavenging ability. Reaction mixtures, containing 0.5 mL of the relevant compound (dissolved in EtOH) and 2.5 mL of a 100 μM DPPH ethanolic solution, were added to 96-well microtiter plates and incubated at 37 °C for 30 min. Absorbances were measured at 515 nm. Percent inhibition was determined by comparison with an EtOH-treated control group. IC_50_ values denote the concentration of samples required to scavenge 50% of the DPPH free radicals.

## 4. Conclusions

Compounds **5** and **6** are new monoterpene phenolic glycosides. Compounds **1**, **3** and **4** were isolated from the *Viburnum* genus for the first time, and compounds **2** and **7** from the *Viburnum sargentii* Koehne for the first time. Compounds **1**–**7** were also assayed for their antioxidant activities with DPPH free radicals, and the data proved that (−)-Epicatechin showed the strongest antioxidant activity.
